# Thiostrepton Suppresses the Progression of Rhabdomyosarcoma by Inhibiting the PI3K‐AKT Signaling Pathway

**DOI:** 10.1002/pdi3.70014

**Published:** 2025-06-29

**Authors:** Yu Wang, Peng Hong, Zhiqiang Gao, Wei Ma, Zaihong Hu, Jie Lin, Kongkong Cui, Qinlin Shi, Xiao‐Mao Tian, Guanghui Wei

**Affiliations:** ^1^ Department of Urology Children's Hospital of Chongqing Medical University National Clinical Research Center for Child Health and Disorders Ministry of Education Key Laboratory of Child Development and Disorders Chongqing Key Laboratory of Structural Birth Defect and Reconstruction Chongqing China

**Keywords:** childhood neoplasms, PI3K‐AKT signaling pathway, rhabdomyosarcoma, thiostrepton (TST)

## Abstract

Rhabdomyosarcoma (RMS), the most common pediatric soft tissue sarcoma with 5‐year survival below 30% in high‐risk/metastatic cases, was investigated through integrated bioinformatics analysis (identifying 269 conserved differentially expressed genes in GEO datasets GSE28511/GSE141690) and experimentally validated thiostrepton (TST), a ribosomal‐targeting antibiotic, as a potent therapeutic candidate via Connectivity Map analysis (*p* < 0.05, score ≈ −1). In‐vitro studies demonstrated TST's dose‐/time‐dependent suppression of RMS proliferation (IC50 4.986–9.764 μmol/L), migration and invasion, G0/G1 cell cycle arrest, and apoptosis induction. In vivo, TST (3.4 mg/mL, 4 weeks) significantly inhibited tumor growth (*p* < 0.05 vs. phosphate buffered saline [PBS]) without organ toxicity. RNA sequencing identified the phosphatidylinositol 3‐kinase/protein kinase B (PI3K‐AKT) pathway as the primary suppressed pathway (False Discovery Rate [FDR] < 0.05), with concurrent downregulation of downstream regulators (*AKT, JAK, CDKs*). This was confirmed by PI3K activator 740 Y‐P rescue experiments, which partially reversed the effects of TST (*p* < 0.05). These findings establish TST as a multi‐mechanism PI3K‐AKT inhibitor for refractory RMS while validating Connectivity Map (Cmap)‐driven drug repurposing for pediatric oncology.

## Graphical abstract

The study establish TST as a multi‐mechanism PI3K‐AKT inhibitor for refractory RMS while validating Cmap‐driven drug repurposing for pediatric oncology.

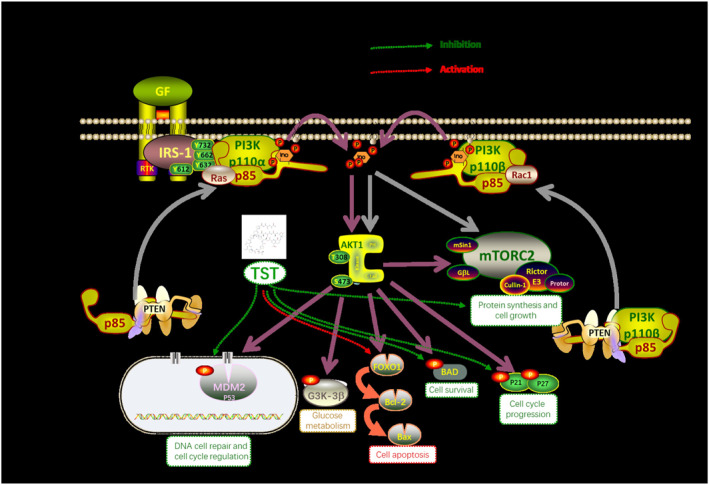

## 1 Introduction

Rhabdomyosarcoma (RMS) is the most common soft tissue sarcoma in children and holds a pivotal position among pediatric malignancies. With an incidence of approximately 4.5 cases per million children, RMS accounts for 5%–10% of all pediatric tumors and 45% of soft tissue sarcomas [1].

RMS is highly heterogeneous and is classified into distinct histological subtypes, including embryonal rhabdomyosarcoma (ERMS) and alveolar rhabdomyosarcoma (ARMS), each exhibiting unique pathogenesis, clinical manifestation, and prognoses [2, 3]. ERMS and ARMS are the predominant subtypes in children. ERMS, which often arises in the head, neck, and genitourinary regions, is associated with a relatively favorable survival rate. In contrast, ARMS tends to affect the extremities or trunk muscles, exhibits stronger treatment resistance, and frequently metastasizes to regional lymph nodes [4].

Over the past few decades, multidisciplinary treatment strategies have significantly improved outcomes for RMS patients, with 5‐year survival rates reaching approximately 70% for low‐risk and some intermediate‐risk cases [5, 6]. However, high‐risk and metastatic RMS patients still face poor therapeutic outcomes, often with 5‐year survival rates below 30% [7, 8, 9]. Additionally, conventional therapies are accompanied by adverse effects such as myelosuppression, which compromise patients' quality of life and long‐term health [10, 11]. Furthermore, the heterogeneous treatment responses across subtypes, the emergence of drug resistance, and the lack of reliable biomarkers for prognosis and treatment prediction hinder the implementation of precise and personalized therapies. These challenges underscore the urgent need to explore novel therapeutic agents.

Thiostrepton (TST), a structurally unique and biologically diverse compound, was first isolated from *Streptomyces azureus* in 1954 and initially used as a veterinary antibiotic for bacterial infections [12]. As a sulfur‐containing peptide antibiotic, its distinctive structure confers exceptional biological activity and mechanisms of action. In oncology research, TST has garnered increasing attention due to its therapeutic potential. For instance, in colorectal cancer, TST stimulates *TRIM6* to downregulate *TIS21/FOXM1* overexpression, thereby inhibiting cancer cell proliferation. In breast cancer, liposome‐encapsulated TST formulations suppress tumor growth. In non‐small cell lung cancer, TST targets cancer stem cells and enhances chemotherapeutic efficacy. In acute lymphoblastic leukemia, TST modulates key genes to inhibit cell growth and induce apoptosis. In high‐grade serous ovarian cancer, TST specifically suppresses *PAX8* to restrict tumor progression [13, 14, 15, 16, 17, 18].

Despite its promising antitumor effects in various cancers, the role and mechanisms of TST in RMS remain unexplored. Investigating the therapeutic potential and underlying mechanisms of TST in RMS holds significant clinical value and may pave the way for novel treatment strategies for this aggressive pediatric malignancy.

## 2 Materials and Methods

### 2.1 Screening of Potential Therapeutic Compounds

Raw data from RMS‐related datasets (GSE28511 and GSE141690) were obtained from the Gene Expression Omnibus (GEO) database (https://www.ncbi.nlm.nih.gov/geo/). After normalization using R software, samples with complete information were selected for analysis. The limma package was used to normalize the expression matrix and identify differentially expressed genes (DEGs, *p* < 0.05 and *|log*_*2*_ *(Fold Change)|* > 1). Common DEGs between the two datasets were compiled and uploaded to the Connectivity Map (Cmap) database (https://clue.io/) in the required format. Using the “Gene Signature Search” module, compounds with *p* < 0.05, negative scores, and values approaching −1 were selected as potential therapeutic candidates.

### 2.2 Cell Culture

The RMS cell lines A204 and RD were cultured in McCoy's 5A medium (supplemented with 10% fetal bovine serum [FBS]) and Dulbecco's modified eagle's medium (DMEM) (supplemented with 10% FBS), respectively. Cells were maintained at 37°C, 5% CO_2_, and 100% humidity, with medium changes every 2–3 days and passaging every 6–7 days.

### 2.3 Cell Proliferation and Half Maximal Inhibitory Concentration (IC50) Determination (Cell Counting Kit‐8 [CCK‐8] Assay)

Log‐phase A204 and RD cells were seeded in 96‐well plates at 10,000 cells/well and incubated for 24 h. Cells were then treated with varying concentrations of TST (0, 0.5, 1, 3, 6, 12, 25, 50, 100 μmol/L, prepared in dimethyl sulfoxide [DMSO]), along with medium and PBS controls. After 24 h, 10 μL of CCK‐8 reagent was added to each well and incubated for 2 h. Absorbance was measured at 450 nm using a microplate reader, and IC50 values were calculated using SPSS and GraphPad Prism.

### 2.4 Cell Proliferation Assay (CCK‐8 Assay)

Log‐phase A204 and RD cells were seeded in 8 × 96‐well plates (10,000 cells/well) and incubated for 24 h. Cells were treated with 5 μmol/L or 10 μmol/L TST, along with DMSO vehicle control, medium‐only, and PBS groups. At 0, 24, 48, and 72 h, 10 μL CCK‐8 reagent was added, followed by 2‐h incubation. Absorbance was measured at 450 nm to assess proliferation.

### 2.5 Wound Healing Assay

Log‐phase A204 and RD cells were adjusted to 5 × 10^5^ cells/mL and seeded in 6‐well plates until 80% confluent. After PBS washing, a 10 μL pipette tip was used to create a scratch. Images were taken to record the initial scratch width. Cells were then treated with 5 μmol/L or 10 μmol/L TST in serum‐free medium, along with DMSO control, and images were captured at 0, 12, and 24 h to measure migration.

### 2.6 Transwell Assay

A 5% Matrigel matrix was prepared in serum‐ and antibiotic‐free medium and applied to Transwell inserts (60 μL/insert). After 3‐h incubation at 37°C, excess liquid was removed, and inserts were hydrated with 100 μL serum‐free medium for 30 min. Serum‐starved cells (1 × 10^6^/mL, 100 μL/insert) were seeded in the upper chamber, whereas the lower chamber contained a complete medium with 5 μmol/L or 10 μmol/L TST or DMSO control. After 24 h, noninvaded cells were removed and invaded cells were fixed with 4% paraformaldehyde, stained with crystal violet and counted under a microscope.

### 2.7 Apoptosis Assay

Log‐phase A204 and RD cells (2 × 10^5^/mL) were seeded in 6‐well plates and treated with 5 μmol/L or 10 μmol/L TST or DMSO control for 24 h. Cells were collected, washed with cold PBS, resuspended in Binding Buffer, and stained with annexin V‐fluorescein isothiocyanate/propidium iodide (Annexin V‐FITC/PI) (5 μL each) for 30 min at 4°C in the dark. Apoptosis was analyzed by flow cytometry.

### 2.8 Cell Cycle Analysis

Log‐phase A204 and RD cells (1 × 10^6^/mL) were seeded in 6‐well plates and treated with 5 μmol/L or 10 μmol/L TST or DMSO control for 24 h. Cells were collected, fixed in 70% ethanol overnight, washed, resuspended in PBS, and stained with PI for 30 min at room temperature in the dark. After filtration through a 300‐mesh sieve, cell cycle distribution was analyzed by flow cytometry.

### 2.9 Animal Model Establishment

Nineteen 4–6 week‐old male BALB/c‐nu mice were housed in a specific pathogen‐free (SPF) facility. Log‐phase RMS cells (5 × 10^7^/mL, 0.1 mL/mouse) were subcutaneously injected into the right flank. Informed by dosages from analogous animal studies, we systematically evaluated anti‐tumor efficacy and safety, determining 17 mg/kg as the optimal in‐vivo dose to balance therapeutic benefit and animal welfare [19]. Mice were divided into 3 groups:

TST group (0.1 mL TST, 17 mg/kg, every 2 days).

Cyclophosphamide (CTX) group (0.1 mL, 20 mg/kg, every 2 days).

PBS control group (0.1 mL PBS, every 2 days).

Treatments lasted 4 weeks, with tumor volume measured every 6 days. After 4 weeks, mice were anaesthetized, blood was collected via orbital puncture, and organs/tumors were excised for histological analysis or frozen storage. Terminal deoxynucleotidyl transferase dUTP nick‐end labeling (TUNEL) staining was performed to assess apoptosis induction, and organ toxicity was evaluated. The study was approved by the Ethics Committee of Children's Hospital, Chongqing Medical University.

### 2.10 Transcriptome Sequencing

A204 cells were treated with TST or DMSO control, washed with cold PBS, and lysed in Trizol for RNA extraction. RNA quality was assessed using Nanodrop 2000 and 1% agarose gel electrophoresis. mRNA was enriched using Oligo(dT) magnetic beads, and libraries were constructed via fragmentation, reverse transcription, and end repair. Library quality was verified using Agilent 2100 Bioanalyzer and quantitative polymerase chain reaction (qPCR). Sequencing was performed on an Illumina platform (PE150 mode), generating ≥ 30 M reads/sample. After quality filtering, reads were aligned to the reference genome using hierarchical indexing for spliced alignment of transcripts 2 (HISAT2), and fragments per kilobase of exon model per million mapped reads (FPKM) values were calculated using StringTie. DEGs (*|log*_*2*_ *(FC)|* ≥ 1, adjusted *p* ≤ 0.05) were identified using differential expression analysis for sequence count data 2 (DESeq2), followed by Gene Ontology (GO), Kyoto Encyclopedia of Genes and Genomes (KEGG), and Gene Set Enrichment Analysis (GSEA) analyses (ClusterProfiler).

### 2.11 Statistical Analysis

Data were analyzed using SPSS 21.0 and GraphPad Prism, presented as mean ± SD. Student's *t*‐test (two groups), one‐way ANOVA (multiple groups), and Spearman correlation were used, with *p* < 0.05 considered statistically significant.

## 3 Results

### 3.1 High‐Throughput Transcriptome Sequencing Predicts TST as a Potential Inhibitor of RMS

**FIGURE 1:**
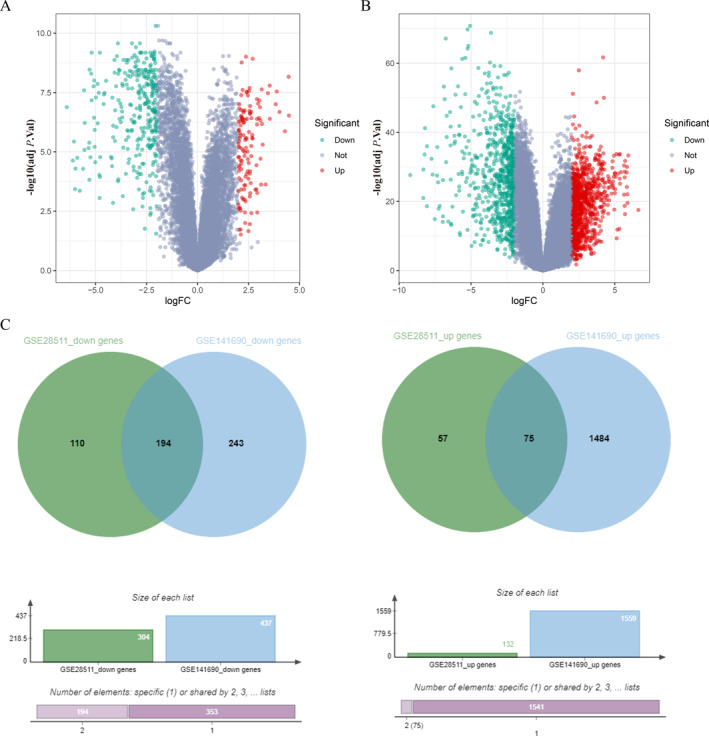
DEGs in RMS GSE28511 and GSE141690, heat map, volcanic map, Venn diagram. (A) DEGs in the RMS gene expression datasets GSE28511. (B) DEGs in the RMS gene expression datasets GSE141690. (C) The common DEGs in RMS GSE28511 and GSE141690. *X*‐axis indicates the fold change (log‐scaled), whereas the *Y*‐axis shows the *p* values (log‐scaled). Each symbol represents a different gene, with red denoting upregulated genes and green indicating downregulated genes based on predefined criteria in A and B. *p* value < 0.05 is considered as statistically significant, whereas fold change = 1.5 is set as the threshold. The overlapping region represents genes significantly upregulated in both datasets, whereas the non‐overlapping regions indicate dataset‐specific upregulated/downregulated DEGs in C. adj *P*. val, adjusted *p* value; DEGs, differentially expressed genes; FC, fold change threshold; RMS, rhabdomyosarcoma.

We analyzed 84 RMS tumor samples and 22 normal muscle tissue samples from the GSE28511 and GSE141690 datasets. Differential expression analysis revealed 436 DEGs (132 upregulated, 304 downregulated) in GSE28511 (Figure 1A) and 1996 DEGs (1559 upregulated, 437 downregulated) in GSE141690 (Figure 1B). Intersection analysis identified 75 commonly upregulated and 194 downregulated genes (Figure 1C).

Using these DEGs as input, we queried the Cmap database to identify compounds that could reverse RMS‐associated gene expression patterns. Among the top candidates (Table 1), several—including CEP‐33779 [20], dasatinib [21], sorafenib [22], oprozomib [23], and refametinib [24]—have shown antitumor effects in human cancers. It is worth noting that TST, a protein synthesis inhibitor, exhibited the strongest negative enrichment score, suggesting its potential to counteract RMS‐associated transcriptional dysregulation.

**TABLE 1:** The top 10 candidate drugs for Cmap analysis.

Rank	Cmap name	Enrich score	Drug category
1	CEP‐33779	−0.52	*JAK* inhibitor
2	Pimozide	−0.52	Dopamine receptor antagonist
3	Dasatinib	−0.52	KIT inhibitor|Bcr‐Abl inhibitor|Ephrin inhibitor|PDGFR inhibitor|Src inhibitor|Tyrosine kinase inhibitor
4	Sorafenib	−0.52	RAF inhibitor|FLT3 inhibitor|KIT inhibitor|PDGFR inhibitor|RET inhibitor|VEGFR inhibitor
5	Thiostrepton	−0.52	Protein synthesis inhibitor
6	Aripiprazole	−0.52	Serotonin receptor agonist|Serotonin receptor antagonist
7	Rilpivirine	−0.52	Non‐nucleoside reverse transcriptase inhibitor
8	PI‐828	−0.52	PI3K inhibitor
9	Oprozomib	−0.52	Proteasome inhibitor
10	Refametinib	−0.52	MAPK/ERK kinase inhibitor

Abbreviations: Bcr‐Abl, breakpoint cluster region‐abelson tyrosine kinase; ERK, extracellular signal‐regulated kinase; FLT3, FMS‐like tyrosine kinase 3; *JAK*, Janus kinase; KIT, KIT tyrosine kinase; MAPK, mitogen‐activated protein kinase; PDGFR, platelet‐derived growth factor receptor; PDGFR, platelet‐derived growth factor receptor; PI3K, phosphatidylinositol 3‐kinase; RAF, rapidly accelerated fibrosarcoma kinase; RET, RET proto‐oncogene tyrosine kinase; VEGFR, vascular endothelial growth factor receptor.

### 3.2 TST Suppresses RMS Proliferation, Migration, and Invasion

**FIGURE 2:**
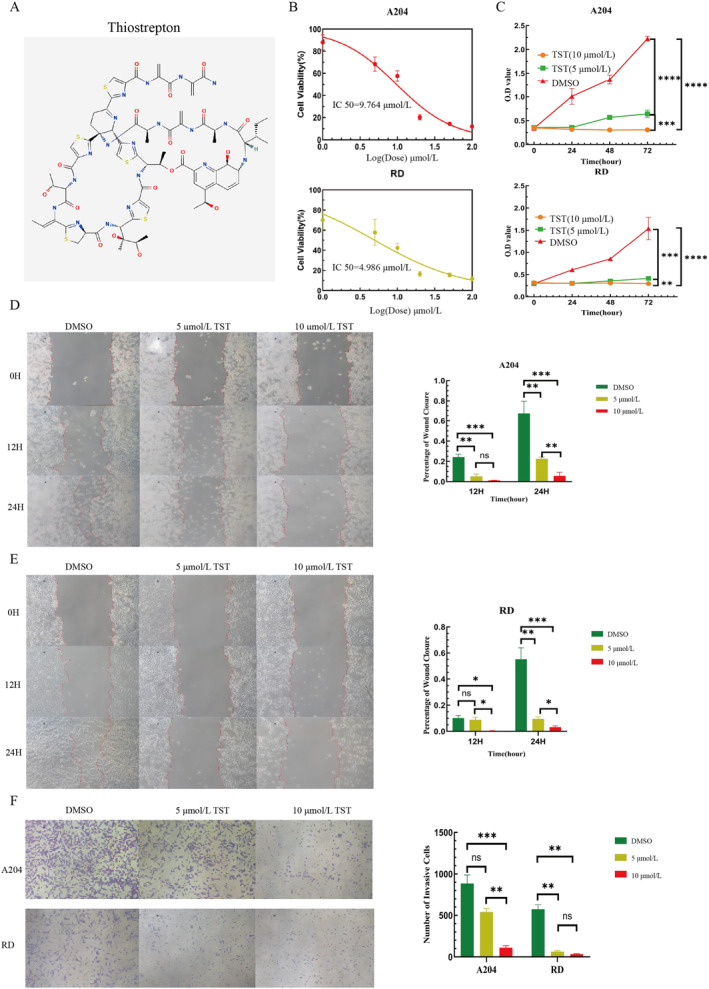
TST reduces the viability and proliferation, migration and invasion of RMS cells. (A) The molecular structure of thiostrepton. (B) A204 and RD cells were treated with TST (0–100 μmol/L, log‐scaled: 0–2) for 24 h, and cell viability was determined using the CCK‐8 assay. (C) O.D. value of the CCK‐8 assay conducted at 0, 24, 48, and 72 h using RMS cells treated with different concentrations of TST (5 or 10 μmol/L) and DMSO. (D, E) Percentage of wound closure of the scratch assay conducted at 0, 12, and 24 h using RMS cells treated with different concentrations of TST (5 or 10 μmol/L) and DMSO. (F) Number of invasive cells of the Transwell invasion assay on RMS cells treated with different concentrations of TST (5 or 10 μmol/L) and DMSO. ns: *p* > 0.05, *: *p* < 0.05, **: *p* < 0.01, ***: *p* < 0.001, ****: *p* < 0.0001. CCK‐8, cell counting kit‐8; DMSO, dimethyl sulfoxide; O.D., optical density; RMS, rhabdomyosarcoma; TST, thiostrepton.

TST, a sulfur‐containing peptide antibiotic, possesses a unique molecular structure (Figure 2A). This structure endows it with the ability to interact specifically with numerous biological macromolecules. Such specific binding serves as the foundation for its efficacy in the field of tumor treatment. Compared with other drugs, TST's chemical structure is more conducive to its penetration through the cell membrane, enabling it to target tumor cells more effectively. Consequently, TST exhibits distinct advantages in the research on the treatment of RMS. This is a crucial reason why we selected it for subsequent research. Given the high proliferative capacity of RMS cells, we first assessed TST's inhibitory effects using CCK‐8 assays. TST dose‐dependently suppressed RMS cell viability, with IC50 values of 9.764 μmol/L (A204) and 4.986 μmol/L (RD) (Figure 2B). Subsequent time‐course experiments confirmed that 5 μmol/L and 10 μmol/L TST significantly inhibited proliferation in a time‐ and concentration‐dependent manner (Figure 2C).

Wound healing assays demonstrated that TST markedly reduced A204 and RD cell migration, with stronger effects at higher concentrations (Figure 2D, E). Similarly, Transwell invasion assays revealed that TST dose‐dependently impaired RMS cell invasiveness (Figure 2F).

### 3.3 TST Induces Cell Cycle Arrest and Apoptosis in RMS

**FIGURE 3:**
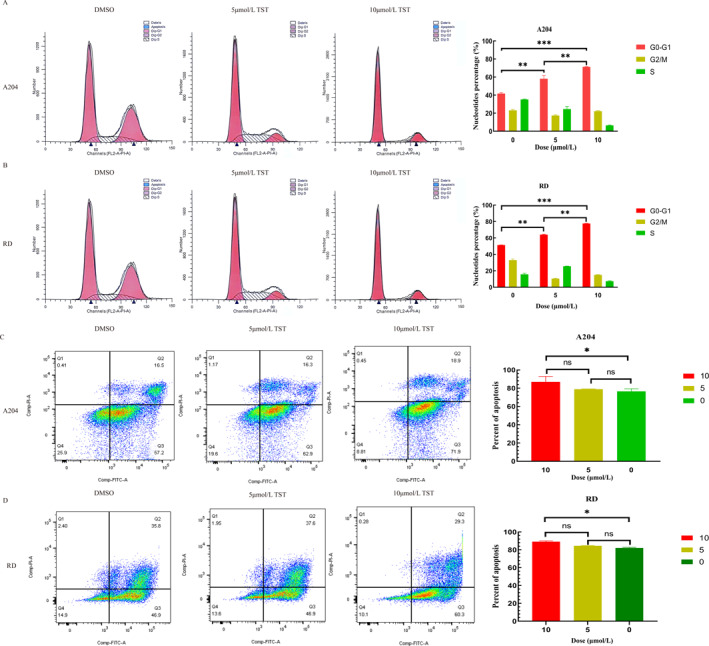
TST induces cell cycle arrest and apoptosis in RMS. (A) Percentage of cells in the G0‐G1 phase of the flow cytometry on A204 cells treated with different concentrations of TST (5 or 10 μmol/L) and DMSO. (B) Percentage of cells in the G0‐G1 phase of the flow cytometry on RD cells treated with different concentrations of TST (5 or 10 μmol/L) and DMSO. (C) Cell apoptosis rate of the flow cytometry on A204 cells treated with different concentrations of TST (5 or 10 μmol/L) and DMSO. (D) Cell apoptosis rate of the flow cytometry on RD cells treated with different concentrations of TST (5 or 10 μmol/L) and DMSO. ns: *p* > 0.05, *: *p* < 0.05, **: *p* < 0.01, ***: *p* < 0.001, ****: *p* < 0.0001. DMSO, dimethyl sulfoxide; RMS, rhabdomyosarcoma; TST, thiostrepton.

Given TST's effects on proliferation and motility, we investigated its impact on cell cycle progression and apoptosis. Flow cytometry showed that TST treatment led to dose‐dependent cell cycle arrest (Figure 3A, B). Additionally, apoptosis assays confirmed that TST significantly increased apoptotic rates in both A204 and RD cells (Figure 3C, D).

### 3.4 TST Inhibits RMS Tumor Growth In Vivo With Favorable Biosafety

**FIGURE 4:**
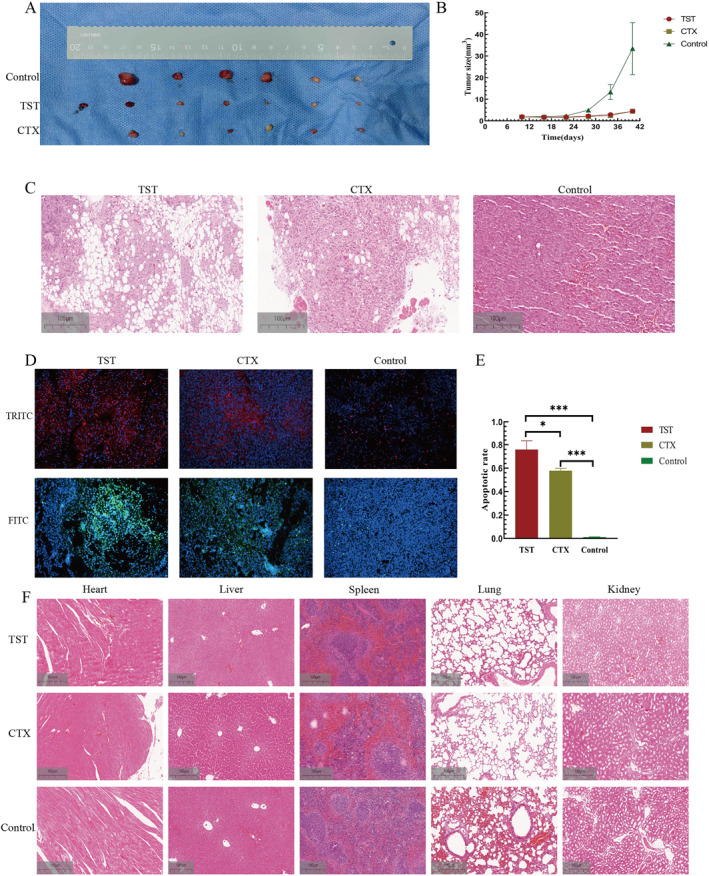
TST inhibitor suppressed RMS growth in vivo. (A) TST, CTX, and control group of tumor‐bearing mice. (B) Tumor size growth curve of tumor‐bearing mice in 3 groups. (C) Effects of TST on tumors in tumor‐bearing mice and the two other groups. (D) TRITC and FITC labeled immunofluorescence TUNEL images of 3 groups. (E) The apoptosis rate histogram of RMS cells in vivo in 3 groups. (F) The toxicity of TST on organs of tumor‐bearing mice of 3 groups. ns: *p* > 0.05, *: *p* < 0.05, **: *p* < 0.01, ***: *p* < 0.001, ****: *p* < 0.0001. CTX, cyclophosphamide; FITC, fluorescein isothiocyanate; TRITC, tetramethylrhodamine; TST, thiostrepton; TUNEL, terminal deoxynucleotidyl transferase dUTP nick‐end labeling.

To evaluate TST's therapeutic potential, we established a xenograft mouse model and treated animals with TST (3.4 mg/mL), cyclophosphamide (CTX, 4 mg/mL), or PBS (control) for 30 days. TST and CTX significantly suppressed tumor growth compared to the control group (*p* < 0.05, Figure 4A, B).

Hematoxylin and Eosin (H&E) staining of tumor tissues revealed reduced mitotic figures and expanded necrotic areas in the TST group, indicating proliferation inhibition (Figure 4C). TUNEL staining further confirmed that TST promoted apoptosis in RMS tumors (*p* < 0.05, Figure 4D, E).

To assess biosafety, we examined heart, liver, spleen, lung, and kidney tissues via H&E staining. No significant organ toxicity was observed in TST‐treated mice (Figure 4F).

### 3.5 TST Exerts Antitumor Effects by Suppressing the Phosphatidylinositol 3‐Kinase/Protein Kinase B (PI3K‐AKT) Pathway

**FIGURE 5:**
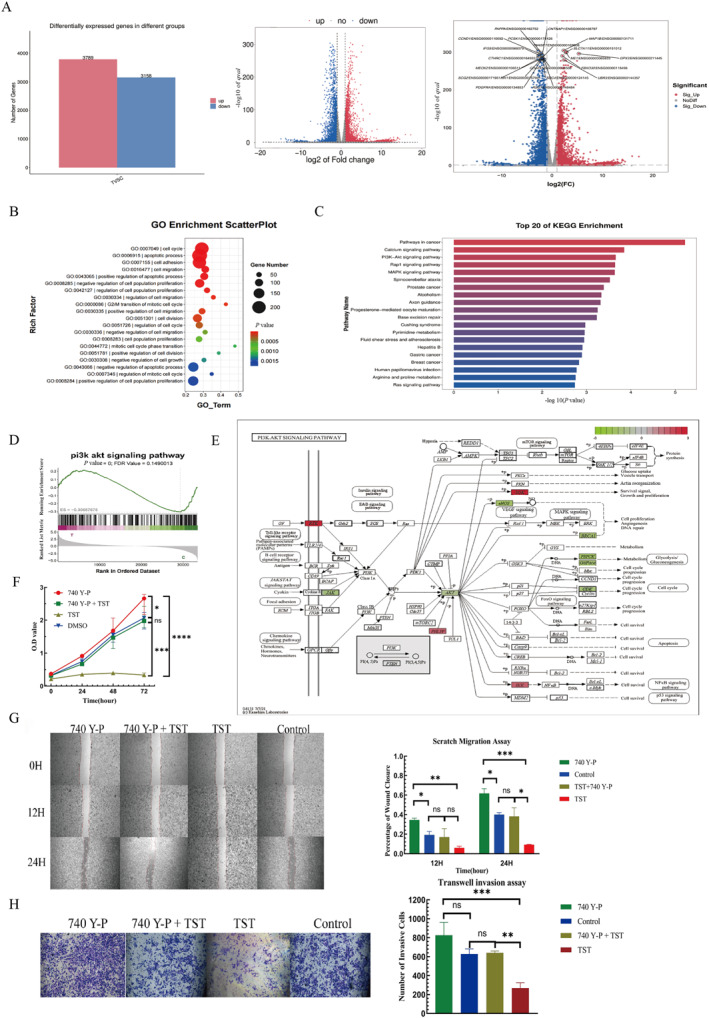
TST exerts antitumor effects by suppressing the PI3K‐AKT pathway. (A) Distribution of differentially expressed genes in RMS cells treated with TST. (B) GO enrichment analysis of cell cycle regulation, apoptosis, adhesion and migration. (C) KEGG enrichment analysis of the top 20 pathways. (D) GSEA enrichment analysis of PI3K‐AKT pathway. (E) PI3K‐AKT signaling pathway diagram. (F) O.D. value of the CCK‐8 assay was conducted at 0, 24, 48, and 72 h using RMS cells treated with different drug groups. (G) Percentage of wound closure of the scratch assay conducted at 0, 12, and 24 h using RMS cells treated with different drug groups. (H) Number of invasive cells of the Transwell invasion assay on RMS cells treated with different drug groups. ns: *p* > 0.05, *: *p* < 0.05, **: *p* < 0.01, ***: *p* < 0.001, ****: *p* < 0.0001. CCK‐8, cell counting kit‐8; GO, gene ontology; GSEA, gene set enrichment analysis; KEGG, kyoto encyclopedia of genes and genomes; O.D., optical density; PI3K‐AKT, phosphatidylinositol 3‐kinase/protein kinase B; RMS, rhabdomyosarcoma; TST, thiostrepton.

To elucidate TST's mechanism, we performed RNA‐sequencing (RNA‐seq) on TST‐treated cells, identifying 6947 DEGs (3789 upregulated, 3158 downregulated) (Figure 5A). GO analysis highlighted enrichment in cell cycle regulation, apoptosis, adhesion, and migration (*p* < 0.01, Figure 5B). KEGG analysis revealed significant suppression of the PI3K‐AKT pathway, a key oncogenic signaling cascade (Figure 5C). GSEA corroborated these findings (Figure 5D).

Further pathway diagram showed that TST downregulated *AKT*, *JAK*, *eNOS*, *BRCA1*, and *CDK*, whereas upregulated *PHLPP*, *IKK*, and *SGK* (Figure 5E). To validate PI3K‐AKT's role, we used the PI3K activator 740 Y‐P, which partially reversed TST's inhibitory effects on proliferation (CCK‐8, *p* < 0.05, Figure 5F), migration (wound healing, *p* < 0.05, Figure 5G), and invasion (Transwell, *p* < 0.05, Figure 5H).

## 4 Discussion

The Cmap database, which establishes drug‐gene associations based on gene expression profiles, has emerged as a powerful tool for elucidating anticancer drug mechanisms and identifying novel therapeutic candidates. By leveraging GEO database‐derived hub genes as search indices within Cmap, researchers can rapidly screen for potential anticancer compounds, accelerating drug discovery and facilitating drug repurposing strategies. This approach has already demonstrated its clinical value, as evidenced by the repurposing of the antiparasitic drug albendazole for hepatocellular carcinoma (NCT04823192), achieving a 46% disease control rate in Phase I trials [25]. Similarly, imatinib—originally developed for chronic myeloid leukemia—has been successfully extended to treat gastrointestinal stromal tumors (GIST) and other kinase‐driven malignancies [26]. Perhaps most remarkably, thalidomide, once withdrawn due to teratogenicity, has been rehabilitated as an effective therapy for multiple myeloma and hematologic cancers [27]. These successes validate the utility of high‐throughput transcriptomics combined with Cmap analysis not only for drug discovery but also for safety assessment—a robust and efficient strategy for therapeutic development.

Our study provides compelling evidence that TST significantly inhibits RMS cell proliferation, migration, and invasion both in vitro and in vivo while promoting apoptosis. These findings align with extensive preclinical research demonstrating TST's broad antitumor activity across multiple cancer types through diverse molecular mechanisms. Moreover, the inhibitory effect of TST on cells exhibits a remarkable dose dependence. As the concentration of TST rises, its inhibitory efficacy on cells gradually strengthens. During the in‐depth analysis of the experimental results, it was discovered that the basal apoptosis levels in the control groups of the A204 and RD cell lines were relatively high. Through analysis, it is hypothesized that certain factors in the cell‐culture process, such as subtle differences in the composition of the culture medium and the culture environment, might have influenced the experimental outcomes. However, the specific impact of these factors on the experimental results remains to be further explored in depth.

In breast, lung, and prostate cancers, TST primarily targets ribosomal function, binding to the 50S subunit to prevent aminoacyl‐tRNA accommodation at the A‐site, thereby disrupting translation initiation/elongation and suppressing oncogenic protein synthesis [28]. Additionally, TST induces ribosomal stress in p53‐wildtype tumors, triggering p53‐dependent cell cycle arrest and apoptosis [29]. It also suppresses key oncogenic pathways including mitogen‐activated protein kinase (MAPK), the mechanistic target of rapamycin (mTOR), and the transcription factor forkhead box protein M1 (FoxM1) [30, 31, 32, 33]. Beyond these effects, TST modulates *Bcl‐2* family proteins and activates endoplasmic reticulum (ER) stress pathways via C/EBP homologous protein (CHOP) upregulation, further amplifying pro‐apoptotic signaling [30, 34].

It is worth noting that our transcriptomic analysis revealed significant suppression of the PI3K‐AKT pathway in TST‐treated RMS cells, with marked downregulation of downstream effectors (*AKT*, *JAK*, *eNOS*, *BRCA1*, *PEPCK*, *G6Pase*, and *CDKs*). This aligns with established paradigms of anticancer drug action—namely, cell cycle disruption, apoptosis induction, and oncogenic pathway inhibition [35, 36, 37]. Crucially, PI3K agonist (740 Y‐P) rescue experiments partially reversed TST's antitumor effects, functionally validating PI3K‐AKT as a key mechanistic target. This pathway's centrality in RMS progression—governing proliferation, survival, metastasis, and therapy resistance—makes it an ideal therapeutic target [38, 39, 40].

It should be emphasized that TST not only inhibits the PI3K‐AKT pathway but also exhibits multi‐target effects. TST can inhibit protein synthesis, disrupting the normal production process of proteins within tumor cells. This interference further affects the growth and proliferation of tumor cells. Meanwhile, TST can regulate FoxM1. As a key transcription factor, FoxM1 plays a vital role in processes such as the proliferation, invasion, and metastasis of tumor cells. TST's regulation of FoxM1 further impacts the biological behavior of tumor cells. These interrelated multi‐target effects collectively contribute to the complex mechanism of action of TST.

In conclusion, our work establishes that TST exerts potent anti‐RMS effects primarily through inhibition of aberrant PI3K‐AKT signaling. Given the pathway's well‐documented role in RMS pathogenesis and treatment resistance, TST represents a promising candidate for further preclinical and clinical evaluation in this aggressive pediatric malignancy. Building on these findings, our future research will focus on elucidating the molecular mechanisms underlying TST‐mediated PI3K‐AKT pathway modulation. We will further explore synergistic therapeutic strategies combining TST with targeted agents (e.g., mTOR inhibitors, mechanistic target of rapamycin inhibitors) or immune checkpoint inhibitors, aiming to enhance antitumor efficacy and overcome drug resistance. Systematic preclinical studies will be conducted to evaluate the safety, pharmacokinetics, and therapeutic potential of TST‐based regimens in advanced RMS models. These efforts aim to provide robust preclinical evidence to support the clinical translation of TST for RMS patients.

## Author Contributions

Y.W. contributed to formal analysis and writing of the original draft. X.‐M.T contributed to the overall research design. G.H.W contributed to supervision. Q.L.S. contributed to reviewing. P.H contributed to the illustrations production. W.M and K.K.C. contributed to data curation. Z.H.H, J.L, and Z.Q.G contributed to the literature collection. All authors read and approved the final manuscript.

## Ethics Statement

The animal use protocol of this study has been reviewed and approved by the Ethics Committee of the Children's Hospital, Chongqing Medical University (CHCMU‐IACUC20250407010).

## Conflicts of Interest

The authors declare no conflicts of interest.

## Data Availability

The datasets used and/or analyzed during the current study are available from the corresponding author on reasonable request.
